# Value of Targeted Biopsies and Combined PSMA PET/CT and mp-MRI Imaging in Locally Recurrent Prostate Cancer after Primary Radiotherapy

**DOI:** 10.3390/cancers14030781

**Published:** 2022-02-03

**Authors:** Marnix Rasing, Marieke van Son, Marinus Moerland, Bart de Keizer, Frank Wessels, Trudy Jonges, Sandrine van de Pol, Wietse Eppinga, Juus Noteboom, Jan Lagendijk, Jochem van der Voort van Zijp, Max Peters

**Affiliations:** 1 Department of Radiation Oncology, University Medical Center Utrecht, Heidelberglaan 100, 3584 CX Utrecht, The Netherlands; m.a.moerland@umcutrecht.nl (M.M.); s.m.g.vandepol-3@umcutrecht.nl (S.v.d.P.); w.s.c.eppinga@umcutrecht.nl (W.E.); j.l.noteboom@umcutrecht.nl (J.N.); j.j.w.lagendijk@umcutrecht.nl (J.L.); j.r.n.vandervoortvanzyp@umcutrecht.nl (J.v.d.V.v.Z.); m.peters-10@umcutrecht.nl (M.P.); 2 Department of Urology, Amsterdam University Medical Center, Meibergdreef 9, 1105 AZ Amsterdam, The Netherlands; m.j.vanson@amsterdamumc.nl; 3 Department of Radiology and Nuclear Medicine, University Medical Center Utrecht, Heidelberglaan 100, 3584 CX Utrecht, The Netherlands; b.deKeizer@umcutrecht.nl (B.d.K.); f.j.wessels-3@umcutrecht.nl (F.W.); 4 Department of Pathology, University Medical Center Utrecht, Heidelberglaan 100, 3584 CX Utrecht, The Netherlands; g.n.jonges@umcutrecht.nl

**Keywords:** radiorecurrent prostate cancer, local recurrence, targeted biopsies, PSMA PET/CT, multiparametric MRI, positive predictive value

## Abstract

**Simple Summary:**

After primary radiotherapy for prostate cancer, patients may develop an isolated local recurrence. The diagnostic workup of these recurrences guides decision making for potential focal salvage treatments. The aim of this study was to determine the positive predictive value (PPV) of combined multiparametric (mp) MRI and prostate specific membrane antigen (PSMA) PET/CT imaging in this setting, with histological conformation using MR-guided targeted biopsies. In 41 patients counseled for focal salvage high dose rate (HDR) brachytherapy, a PPV of 97.6% was found for combined mp-MRI and PSMA PET/CT. Therefore, biopsies can safely be omitted in these patients.

**Abstract:**

Radiorecurrent prostate cancer is conventionally confirmed using systematic and/or targeted biopsies. The availability of multiparametric (mp) MRI and prostate specific membrane antigen (PSMA) PET/CT has increased diagnostic accuracy. The objective was to determine the positive predictive value (PPV) of combined mp-MRI and PSMA PET/CT and whether pathology verification with MR-targeted biopsies remains necessary for patients with radiorecurrent prostate cancer. Patients with locally recurrent prostate cancer who were referred for 19 Gy single-dose MRI-guided focal salvage high dose rate (HDR) brachytherapy between 2015 and 2018 were included in the current analysis. Patients were selected if they underwent pre-biopsy mp-MRI and PSMA PET/CT. Based on these images, lesions suspect for isolated tumor recurrence were transperineally biopsied using transrectal ultrasound fused with MRI. A total of 41 patients were identified from the database who underwent cognitive targeted (*n* = 7) or MRI/PSMA-transrectal ultrasound (TRUS) fused targeted (*n* = 34) biopsies. A total of 40 (97.6%) patients had positive biopsies for recurrent cancer. Five patients initially had negative biopsies (all MRI/PSMA-TRUS fusion targeted), four of whom recurrence was confirmed after a re-biopsy. One (2.4%) patient refused re-biopsy, leading to a positive predictive value (PPV) for combined imaging of 97.6%. Biopsies can therefore safely be withheld when the results of the combined mp-MRI and PSMA PET/CT are conclusive, avoiding an unnecessary invasive and burdensome procedure.

## 1. Introduction

Biochemical recurrences after primary radiotherapy occur in 5–54% of patients [1,2,3,4], and in approximately 52% of patients with increasing prostate specific antigen (PSA) levels a local recurrence is present [5]. Often, men with recurrent disease are being treated with (deferred) androgen deprivation therapy (ADT) [6,7]. This is a palliative treatment with a temporary suppressive effect; moreover, it is associated with side effects that impact quality of life of these patients. Radiorecurrent prostate cancer is often confined to the prostate [8] and mostly located focally at the site of the initially largest and/or highest grade index lesion [9,10,11,12]. Consequently, an opportunity for focal salvage therapy arises [13,14], which is targeted to the tumor focus within the prostate. With focal treatment as opposed to whole-gland treatment, the surrounding healthy tissue can be better spared, thereby decreasing the risk of serious toxicity while offering a potentially curative treatment. Characteristics such as age, PSA doubling time, tumor volume, and presence of late genitourinary or gastrointestinal radiation toxicity should be taken into account when selecting eligible patients for focal salvage treatment and for ruling out patients who will not benefit from local treatment. Traditionally, pathological confirmation has had a key role in confirming disease and guiding treatment strategy in prostate cancer and remains pivotal in the primary setting [15]. In the radiorecurrent setting, both imaging and pathologic confirmation modalities are available for the clinical workup. However, histological confirmation is an invasive procedure. Although an upgrade in Gleason score compared to the primary setting might be encountered [10], a re-biopsy mostly provides information on the presence or absence of adenocarcinoma, since the Gleason grading system is not always appropriate for scoring radiorecurrent lesions due to treatment effects [10,16,17]. A less-invasive alternative for detecting local recurrence is a combination of imaging with multiparametric (mp) MRI and prostate specific membrane antigen (PSMA) PET/CT. Both modalities have gained an important role in staging and restaging of prostate cancer, as well as in treatment planning [5,18,19,20,21,22,23,24].

The main aim of this study is to determine the positive predictive value of combined mp-MRI and PSMA PET/CT imaging and to determine the added value of targeted biopsies in the clinical workup towards focal salvage treatment of locally recurrent prostate cancer.

## 2. Materials and Methods

Our institutional review board approved this study. The ‘no objection’ regulation of our institute for secondary use of tissue was applicable. All patients also received an information leaflet and signed informed consent forms prior to inclusion.

### 2.1. Study Population

Patients with increasing PSA-values at least 2 years after primary radiotherapy (low dose rate (LDR) brachytherapy or external beam radiotherapy) for prostate cancer, who had subsequent diagnostic imaging with ^68^Ga PSMA PET/CT and 3T mp-MRI followed by MR-guided targeted biopsies were included prospectively. Suspect prostate lesions were transperineally biopsied using either combined MRI/PSMA-targeted cognitive biopsies or targeted MRI/PSMA-TRUS fused (transrectal ultrasound) biopsies. In case more than 1 PSMA PET/CT scan was performed in the diagnostic workup, the most recent scan was used. Focal salvage HDR brachytherapy was performed from July 2015 to January 2018, with an MR-guided method and a single 19 Gy dose on locally recurrent cancer in prostate and/or seminal vesicles. PSMA PET/CT and mp-MRI images were fused and used to delineate the gross tumor volume (GTV) on the MRI. The treatment procedure has been described previously [25].

### 2.2. Variables

Patient and disease characteristics were collected and analyzed, including: Gleason score of primary tumor and of local recurrence; carcinoma volume across all biopsy cores; side and number of positive biopsy cores; PSMA-receptor density in biopsy tissue; maximum body weight corrected standardized uptake value (SUV) on PSMA PET/CT; maximum tumor diameter, localization and mean apparent diffusion coefficient (ADC) on MRI; age; initial prostate-specific antigen level (iPSA) before primary radiotherapy treatment; PSA before focal salvage HDR treatment; and prostate cancer TNM stage.

For the purpose of analyses of the tissue, per patient, 3 slices of 4 μm were cut of every paraffin-embedded tissue-sample of tumor-positive biopsy material. PSMA-receptor density in biopsy tissue was determined with immunohistochemistry and assessed by an expert uropathologist at our academic center. The maximum body weight corrected SUV on PSMA PET/CT was measured by an expert uroradiologist specialized in nuclear medicine. The maximum tumor diameter, localization, and ADC value on MRI were assessed by another expert uroradiologist.

### 2.3. Image Acquisition and Analysis

^68^Ga-PSMA-11 was administered intravenously in a dose of 1.5–2 MBq/kg. Combined PET and CT images from the skull vertex to the thighs were performed approximately 60 min after injection. PET was acquired according to the European Association of Nuclear Medicine recommendations [26]. For SUV measurements, the body weight corrected values were used. PET scans were scored visually and focal increased uptake in the prostate was considered suggestive for recurrent disease. For MRI analysis PI-RADS Prostate Imaging—Reporting and Data System: 2015, Version 2 was used [27]. PI-RADS scores 4 and 5 were considered positive. 

### 2.4. Analyses

For our primary outcome, the positive predictive value of combined PSMA PET/CT and mp-MRI imaging for the presence of recurrent prostate cancer was calculated with a crosstab. All patients with a suspect lesion on imaging and a positive biopsy were divided by the sum of all patients with a suspect lesion on imaging and a positive biopsy and all patients with a suspect lesion on imaging and a negative biopsy. In this regard, the results of targeted prostate biopsies were considered the reference standard. 

Secondary outcomes consisted of: PSMA-receptor density in biopsy tissue, correlation between imaging parameters (maximum SUV on PSMA PET/CT, mean-ADC on MRI, maximum tumor diameter on MRI) and biopsy parameters (PSMA-receptor density, Gleason score/AJCC Histopathological Grade Group [28], and carcinoma volume across all biopsy cores).

### 2.5. Statistical Analysis

Analyses were performed using SPSS version 25.0 (IBM Corp, IBM SPSS Statistics for Windows, Armonk, NY, USA). The Mann-Whitney U test was used to test for differences in both maximum SUV and carcinoma volume across all biopsy cores, in lower versus higher Gleason scores. Correlations between a continuous variable and an ordinal variable were calculated with binary logistic regression analysis or ordered logistic regression. When comparing two continuous variables, a scatter plot was generated in order to determine if linearity was present. The Pearson or Spearman correlation coefficient was determined for linearly and non-linearly correlated variables, or for correlations between two ordinal variables.

## 3. Results

### 3.1. Primary Outcome

A total of 41 patients with suspected local recurrent prostate cancer had both a PSMA PET/CT and mp-MRI visible recurrent tumor and subsequently underwent targeted biopsies. One patient participated twice, as he developed a second local recurrence in a different location after focal salvage HDR brachytherapy and received a second HDR salvage treatment two years later. The mean time between primary and salvage treatment was 7.70 years (SD 3.56) and median PSA before salvage treatment was 4.6 ng/mL (IQR 2.7–7.8, range 1.0–39.0). An overview of baseline characteristics can be found in Table 1 and pre-salvage biopsy and imaging characteristics are presented in Table 2 and Table 3. All patients had a suspected lesion on both PSMA PET/CT and mp-MRI. Biopsies were cognitive targeted (*n* = 7) or MRI-TRUS fusion targeted (*n* = 34). A total of 36 (87.8%) patients had positive biopsies for cancer at first attempt. Four patients were initially negative (all MRI-TRUS fusion targeted) and were re-biopsied, after which recurrent prostate cancer was verified. One (2.4%) patient had negative biopsies and refused re-biopsy. He had a suspect lesion in the base of the prostate on both imaging modalities, a PSA-value of 7.80 ng/mL and was the only patient that ultimately was not treated with focal salvage HDR brachytherapy. Subsequently, his PSA has increased slowly and his preference to refrain from active treatment remains. These five patients with initially negative biopsies showed some differences compared to the remaining patients: they had a lower mean PSMA SUV (6.76 versus 10.1), none had seminal vesicle involvement, and only one of them had a PI-RADS 5 classification on the MRI of recurrent disease, compared to 19 out of 36 in patients with initially positive biopsies. Tumor diameter and gross tumor volume were comparable in the two groups.

The combined mp-MRI and PSMA PET/CT had a positive predictive value for detecting recurrent prostate cancer of 97.6%. Concordance between imaging and biopsies is represented in Table 4, including laterality of the lesions. Concerning the concordance between mp-MRI and PSMA PET/CT: all patients had concordance in tumor location within the prostate and/or seminal vesicle. Regarding laterality, two patients had a unifocal lesion on one imaging modality, when the other modality showed bilateral involvement. Due to very close proximity and overlap, this resulted in expansion of the target volume of the same lesion in the delineation. One patient had a true multifocal recurrence. All others showed concordance in both modalities. 

### 3.2. Correlations between Imaging and Biopsy Parameters

PSMA-receptor density in pre-salvage biopsy tissue was classified as high (*n* = 31, 75.6%) or medium (*n* = 3, 7.3%), see Table 2. 

**Figure 1:**
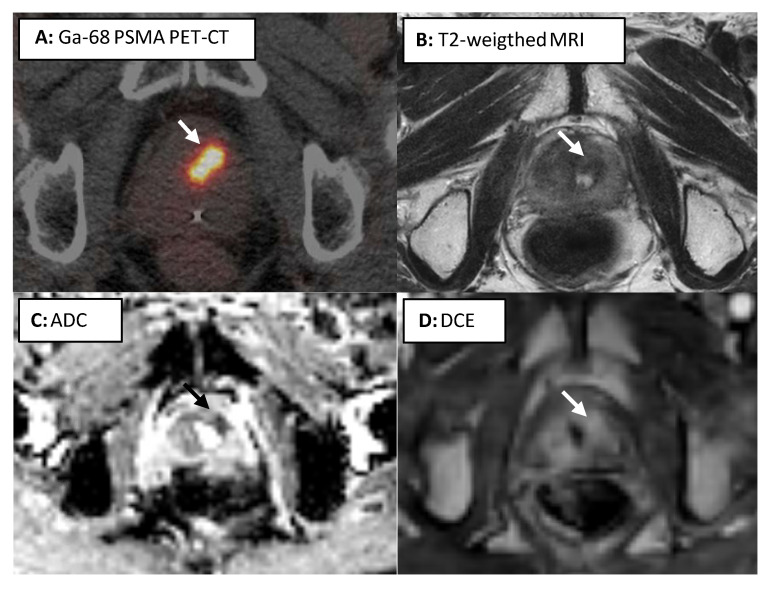
Example of an 82-year old patient who underwent external beam radiotherapy in 2010 with a total dose of 78 Gy in 39 fractions for a cT1cNxMx adenocarcinoma of the prostate, Gleason 3 + 4 = 7, PSA 8.8 ng/mL. He achieved a PSA nadir of 0.4 ng/mL in March 2013. In 2016, his PSA had risen to 3.3 ng/mL. A Gallium (Ga)-68 PSMA PET/CT scan showed a recurrence in the left mid-glandular anterior part of the prostate (Picture **A**, central activity due to urine in the urethra). This was verified on the T2-weighted image (low signal, picture **B**). Diffusion restriction was seen on the apparent diffusion coefficient (ADC) map (**C**) and contrast enhancement was seen on the dynamic contrast enhanced (DCE) series (**D**). He underwent MRI-targeted biopsies of this lesion which showed a recurrence in 7% of the biopsy volume in 1/5 cores, Gleason 3 + 4 = 7. He successfully underwent MRI-guided focal salvage HDR brachytherapy in September 2016. His PSA reached a nadir of <0.1 ng/mL, and slowly started to rise after 2 years, with a peak after 4 years followed by a decrease. No further diagnostics have been performed as of yet. Apart from transient urinary incontinence, no toxicity was reported.

When looking for possible associations between imaging and biopsy parameters and other previously mentioned variables, the following significant associations were found: a higher carcinoma volume across all biopsy cores with a higher Gleason score (AJCC group 1 (*n* = 8) versus 2–5 (*n* = 27): median 6.5% (IQR 5–37.5%) versus 40% (IQR 20–70%), *p* = 0.03) and a higher maximum tumor diameter on MRI with a higher maximum SUV (*p* < 0.01, correlation coefficient 0.516). We further observed higher PSMA SUV in patients with Gleason sum score 7–9 (*n* = 27) compared to 6 (*n* = 8): median 10.1 (IQR 5.9–16.1) versus 4.9 (IQR 4.2–6.4, *p* = 0.01). An example of the combined imaging modalities is shown in Figure 1. No significant correlation was found between maximum SUV and mean-ADC, and between imaging parameters maximum SUV or mean-ADC and biopsy parameters Gleason score/AJCC group. Due to lack of distinctiveness in PSMA-receptor density in biopsy tissue because almost all patients were classified as high density, this variable could not be used for determining possible correlations.

## 4. Discussion

Our study results demonstrate that in patients with increasing PSA-values at least two years after primary radiotherapy for prostate cancer, combined imaging with mp-MRI and PSMA PET/CT has a positive predictive value of 97.6% to find recurrent prostate cancer. Therefore, targeted biopsies have limited added value in the clinical workup towards focal salvage treatment in radiorecurrent prostate cancer.

Prostate cancer is one of the leading types of cancer globally [29], and a substantial number of high-risk patients still develops a biochemical recurrence after primary radiotherapy [1,2,3,4]. The diagnostic workup of suspected isolated local radiorecurrent prostate cancer concerns a significant number of patients, especially since focal salvage strategies have increasingly gained interest in recent years [13,14]. Not all patients will benefit from salvage therapy, considering life expectancy and the sometimes long natural course of recurrent disease [30]. Ideally, diagnostic evaluation of (recurrent) disease leads to a high probability of disease presence or absence, without the use of tests that provide limited additional information yet give rise to potential patient discomfort or complications. Before the introduction of modern imaging modalities, the detection accuracy of locally recurrent prostate cancer was limited; therefore, pathological confirmation was warranted [31]. More recently, mp-MRI and PSMA PET/CT have gained a significant role in the diagnosis and imaging of locally recurrent disease [5,18,19,20,21,22,23,24], PSMA PET/CT also being pivotal for excluding distant metastases. However, current European Society of Medical Oncology (ESMO) guidelines still mention the need for histological confirmation before focal salvage treatment [32].

In the diagnostic setting before primary treatment of local disease, PPVs between 36 and 100% for PSMA PET/CT [33,34,35,36,37], 34 and 85% for mp-MRI [33,34,35,38], and 67 and 85% [35,39] for combined PSMA PET/CT and mp-MRI are reported. Sensitivity and specificity rates between 64 and 95% and 71 and 95% have been found for PSMA PET/CT and between 67 and 96% and 81 and 100% for PSMA PET/MRI in primary prostate cancer detection [40,41]. For mp-MRI alone, sensitivity rates of 43–96% and specificity rates of 23–98% have been described elsewhere [38,40,41]. 

Since radiorecurrent prostate cancer usually appears as a focal in-field recurrence [9,10,11,12], a higher PPV is expected for combined imaging with PSMA PET/CT and mp-MRI in patients with increasing PSA-values after primary radiotherapy compared to the primary pretreatment setting. In addition, postradiation fibrotic changes in prostate tissue (such as acinar distortion, stromal fibrosis, and atrophy) might increase the contrast between benign and malignant tissue on MRI, especially in dynamic contrast enhanced (DCE) and diffusion-weighted imaging (DWI) series [42]. 

To the best of our knowledge, there are no previous studies focusing on the accuracy of combined PSMA PET/CT and mp-MRI imaging in the radiorecurrent setting, with MR-targeted histopathological confirmation available. Previous studies have examined the diagnostic accuracy of PSMA PET/CT or mp-MRI in recurrent prostate cancer, but not in the same setting as in our study, because of differences in primary treatment (all or a subgroup of patients with status after primary prostatectomy or HIFU), location of biopsies (i.e., biopsies of regional or distant metastases), biopsy type (not MR-targeted), or imaging (not a combination of PSMA PET/CT and mp-MRI) [17,36,40,43,44,45,46,47,48].

In a retrospective study, two radiologists examined the accuracy of four different mp-MRI imaging data sets in 53 patients with suspected radiorecurrent prostate cancer, using TRUS-guided biopsies as a reference standard. Thirty-five of them had histologically proven locally recurrent disease. PPV values up to 0.765 were found on a per sextant basis, and up to 0.944 on a per patient basis for detection of prostate cancer for mp-MRI in radiologist 1 and 2 [17]. Groenendaal et al. developed a statistical model in the primary setting that predicts tumor presence on a voxel level, using ADC and DCE MRI images [49]. A mean area under the curve (AUC) of 0.89 was found. We expect a further increase due to the addition of PSMA PET/CT and possibly a further increase in the setting of local recurrent disease such as in the current study as opposed to the primary setting, which our results suggest. Another study examined the diagnostic value of PET/CT imaging with the ^68^Ga-labelled PSMA ligand HBED-CC in the diagnosis of recurrent prostate cancer. Most of these patients had prostatectomy as primary treatment. In the 42 patients where histological confirmation was available, lesion-based PPV was 100%. However local, regional, and distant lesions were taken together and it was not stated how many of the biopsied patients had radiotherapy as primary treatment [44].

Of the five patients in our study who had a negative first biopsy, one patient refused a rebiopsy, and the other four patients had a positive rebiopsy. Possibly, a rebiopsy would have been positive in the other patient, increasing the PPV to 100%. In case the pathologist is able to determine a reliable Gleason score in the radiorecurrent setting, a change in Gleason score might be found. This could be of prognostic interest, but does not influence the treatment strategy. Obtaining (re)biopsies, which is a burdensome and uncomfortable procedure, therefore seems obsolete. Omitting them leads to less patient discomfort, less morbidity such as infectious complications, hematuria, or pain [50], increased availability of health care staff and acceleration of the diagnostic workup, thereby reducing costs. Although both mp-MRI and PSMA PET/CT are accompanied by their own costs, they are a requisite for performing focal salvage. There still might be situations where targeted biopsies can have added value in the diagnostic workup, for instance in case of a substantially increased PSA-value together with a low or ambiguous suspicion of locally recurrent disease on mp-MRI and PSMA PET/CT, or in case of a disconcordance between mp-MRI and PSMA PET. We encourage the use of targeted biopsies in such circumstances where diagnostic uncertainty remains. 

Found correlations between variables were weak, overall we did not find prognostic parameters in this cohort. Higher SUV in tumors with higher Gleason scores have been described previously [51,52]. The association between Gleason score and outcome after focal salvage is not clear: some studies find a correlation [53], but other studies do not [54,55]. Prediction models with a good performance are available, also in the setting of focal salvage HDR brachytherapy [56], in which other parameters have been described to be predictive of biochemical failure after focal salvage HDR brachytherapy for locally recurrent prostate cancer, such as gross tumor volume, pre-salvage PSA-value, and pre-salvage PSA doubling time [56]. Gleason score seems to be of limited added value, due to the inconclusive findings in the literature. 

A strength of this study is that we used PSMA PET/CT and mp-MRI, which are currently the best imaging modalities in case of recurrent and primary disease. Since the biopsies used for histological confirmation were targeted, they can validly be used as reference standard, without risk of bias due to disconcordance between localization of imaging lesions and localization of biopsies, as could result from systematic biopsies [17]. Furthermore, a dedicated uropathologist graded the biopsy specimens.

A limitation could be the single-center nature of this study. However, multiple regional and national hospitals refer to our center for the counseling of focal salvage HDR brachytherapy. Furthermore, although all eligible patients in the timespan of the study participated, others were excluded, for example when they had biopsies prior to the PSMA PET/CT, non-targeted biopsies, or no biopsies at all. Therefore, spectrum bias cannot be ruled out, meaning that diagnostic performance of combined PSMA PET/CT and mp-MRI could be different when used in a different clinical setting. However, in the situation of false-negative results on combined imaging (with positive biopsies), focal salvage treatment would not be feasible because a clear target on imaging is crucial for the execution of the salvage. The small sample size is another limitation. With a larger sample size, more correlations between imaging characteristics and prognostic factors could possibly be found.

Future research could be directed at confirming our results in similar cohorts or could focus on the role of biopsies in patients with local recurrences after focal salvage treatment, for whom a second focal salvage treatment is considered. This is a new, small, but expanding subgroup of patients that is not adequately covered in our study to infer conclusions on. Integrated PET/MRI imaging also seems promising and might be used in radiorecurrent prostate cancer [40,57]. 

## 5. Conclusions

In patients with biochemical recurrence after primary radiotherapy for localized prostate cancer, combined mp-MRI and PSMA PET/CT has a positive predictive value for the detection of a local recurrence of 97.6%. In these patients, targeted biopsies can, therefore, safely be withheld from the workup towards focal salvage HDR-brachytherapy when results of combined mp-MRI and PSMA PET/CT are conclusive, avoiding an unnecessary invasive and burdensome procedure. Our study results can also be of value for patients where other focal salvage strategies are considered, such as cryotherapy or HIFU. The omission of targeted biopsies might also accelerate diagnostic workup and reduce costs. 

## Figures and Tables

**Table 1:** Baseline characteristics.

		No. Patients (%)
Total no. patients		41 (100)
Age (at focal salvage HDR) ^a^		72 (67–76)
iPSA before primary RT ^a,b^		12.4 (8.1–23.8)
PSA before focal salvage HDR ^a,b^		4.6 (2.7–7.8)
Primary prostate cancer treatment	EBRT	21 (51.2)
	I-125 LDR	19 (46.3)
	Focal HDR-BT	1 (2.4)
Gleason score ^c^ primary tumor	3 + 3 = 6	22 (53.7)
	3 + 4 = 7	11 (26.8)
	4 + 3 = 7	5 (12.2)
	Sumscore 8	1 (2.4)
	Sumscore 9/10	2 (4.9)
Time to salvage treatment (years) ^d^		7.70 ± 3.56

^a^: Expressed as median with interquartile range. ^b^: In ng/mL. ^c^: Grouped according to AJCC grade group system. ^d^: Expressed as mean ± standard deviation. HDR: high dose rate. iPSA: (initial) prostate specific antigen. RT: radiotherapy. EBRT: external beam radiotherapy. LDR: low dose rate. BT: brachytherapy.

**Table 2:** Pre-salvage biopsy characteristics.

		No. Patients (%)
Target biopsy type	Cognitive	7 (17.1)
	MRI/PSMA-TRUS fusion	34 (82.9)
Median number of positive cores		3
Positive cores side	Right	19 (46.3)
	Left	14 (34.1)
	Bilateral (midline)	6 (14.6)
	Bilateral (multifocal)	1 (2.4)
	No positive cores	1 (2.4)
Median carcinoma volume across biopsy cores		40%
Gleason score ^a^	3 + 3 = 6	8 (19.5)
	3 + 4 = 7	11 (26.8)
	4 + 3 = 7	11 (26.8)
	Sumscore 8	1 (2.4)
	Sumscore 9/10	4 (9.8)
	Unknown	6 (14.6)
PSMA expression ^b^	Medium	3 (7.3)
	High	31 (75.6)
	Unknown	7 (17.1)
Extra systematic biopsies		3 ^c^ (7.3)
Median number of cores		8
Positive cores side	Righ	1 (33.3)
	No positive cores	2 (66.7)
Concordance positive side between targeted and systematic		1 (33.3)
Gleason score Sumscore 9/10		1 (33.3)

^a^: Grouped according to AJCC grade group system. ^b^: PSMA-receptor density in biopsy tissue. ^c^: In all 3 patients the systematic biopsies were performed in the same procedure as the targeted biopsies. TRUS: transrectal ultrasound. PSMA: prostate specific membrane antigen.

**Table 3:** Pre-salvage imaging characteristics.

		No. Patients (%)
MRI		
Median prostate size (mL) ^a^		28.3 (25.0–37.7)
Median tumor diameter (mm) ^a^		13.0 (10.0–19.5)
Tumor location	Base	7 (17.1)
	Midgland	11 (26.8)
	Apex	6 (14.6)
	Overlapping	4 (9.8)
	Seminal vesicle	5 (12.2)
	Prostate and seminal vesicle	8 (19.5)
Tumor side	Left	11 (26.8)
	Right	18 (43.9)
	Bilateral (midline)	9 (22.0)
	Bilateral (multifocal)	3 (7.3)
PI-RADS	2	1 (2.4)
	3	4 (9.8)
	4	16 (39.0)
	5	20 (48.8)
PSMA PET/CT		
Median SUV max ^a^		6.9 (4.8–12.1)
Tumor location	Base	7 (17.1)
	Midgland	11 (26.8)
	Apex	6 (14.6)
	Overlapping	4 (9.8)
	Seminal vesicle	5 (12.2)
	Prostate and seminal vesicle	8 (19.5)
Tumor side	Left	13 (31.7)
	Right	18 (43.9)
	Bilateral (midline)	7 (17.1)
	Bilateral (multifocal)	3 (7.3)
Imaging TNM stage		
T	T2	29 (70.7)
	T3	12 (29.3)
N	N0	41 (100)
M	M0	39 (95.1)
	M1	2 (4.9)

^a^: Expressed as median with interquartile range. PI-RADS: Prostate Imaging—Reporting and Data System. PSMA: prostate specific membrane antigen. SUV: Standard Uptake Value. TNM: TNM Classification of Malignant Tumors.

**Table 4:** Concordance between biopsies and imaging.

		Biopsy + ^a^			Biopsy −	Total
		Left	Right	Bilateral		
Imaging + ^b^	Left	11	0	0	0	11
	Right	0	17	0	1	18
	Bilateral	3	2	7	0	12
Imaging −		N/A			N/A	
Total		14	19	7	1	41
PPV combined imaging	40/(40 + 1) = 97.6%					

^a^: In the case of a second biopsy after a negative biopsy at the first attempt, data of the second biopsy were used. ^b^: Data of PSMA PET/CT and MRI were combined. N/A: not applicable. PPV: positive predictive value.

## Data Availability

Data available on request due to restrictions, e.g., privacy or ethical. The data presented in this study are available on request from the corresponding author. The data are not publicly available due to regulations regarding the supply of study related material and results as mentioned on the application form used for obtaining pathology samples.
